# Strong Hearts, healthy communities: a rural community-based cardiovascular disease prevention program

**DOI:** 10.1186/s12889-016-2751-4

**Published:** 2016-01-28

**Authors:** Rebecca A. Seguin, Galen Eldridge, Meredith L. Graham, Sara C. Folta, Miriam E. Nelson, David Strogatz

**Affiliations:** 1 Division of Nutritional Sciences, Cornell University, Savage Hall, Room 412, Ithaca, NY 14853 USA; 2 Montana State University Extension, 235 Culbertson Hall, Bozeman, MT 59718 USA; 3 Division of Nutritional Sciences, Cornell University, Savage Hall, Room 413, Ithaca, NY 14853 USA; 4 Friedman School of Nutrition Science and Policy, Tufts University, 150 Harrison Avenue, Boston, MA 02111 USA; 5 Center for Rural Community Health, Bassett Research Institute, One Atwell Road, Cooperstown, NY 13326 USA

**Keywords:** Cardiovascular disease, Rural, Community, Nutrition, Diet, Exercise, Physical activity, Civic engagement, Health promotion, Evidence-based

## Abstract

**Background:**

Cardiovascular disease is the leading cause of death in the United States and places substantial burden on the health care system. Rural populations, especially women, have considerably higher rates of cardiovascular disease, influenced by poverty, environmental factors, access to health care, and social and cultural attitudes and norms.

**Methods/Design:**

This community-based study will be a two-arm randomized controlled efficacy trial comparing a multi-level, community program (Strong Hearts, Healthy Communities) with a minimal intervention control program (Strong Hearts, Healthy Women). Strong Hearts, Healthy Communities was developed by integrating content from three evidence-based programs and was informed by extensive formative research (e.g. community assessments, focus groups, and key informant interviews). Classes will meet twice weekly for one hour for 24 weeks and focus on individual-level skill building and behavior change; social and civic engagement are also core programmatic elements. Strong Hearts, Healthy Women will meet monthly for hour-long sessions over the 24 weeks covering similar content in a general, condensed format. Overweight, sedentary women 40 years of age and older from rural, medically underserved communities (12 in Montana and 4 in New York) will be recruited; sites, pair-matched based on rurality, will be randomized to full or minimal intervention. Data will be collected at baseline, midpoint, intervention completion, and six-month, one-year, and eighteen months post-intervention. The primary outcome is change in body weight; secondary outcomes include physiologic, anthropometric, behavioral, and psychosocial variables. In the full intervention, engagement of participants’ friends and family members in partnered activities and community events is an intervention target, hypothesizing that there will be a reciprocal influence of physical activity and diet behavior between participants and their social network. Family members and/or friends will be invited to complete baseline and follow-up questionnaires about their health behaviors and environment, height and weight, and attitudes and beliefs.

**Discussion:**

Strong Hearts, Healthy Communities aims to reduce cardiovascular disease morbidity and mortality, improve quality of life, and reduce cardiovascular disease-related health care burden in underserved rural communities. If successful, the long-term goal is for the program to be nationally disseminated, providing a feasible model to reduce cardiovascular disease in rural settings.

**Trial registration:**

ClinicalTrials.gov Identifier: NCT02499731 Registered on July 1, 2015.

## Background

Despite declines in heart disease mortality in the United States since 2000, it remains the leading cause of mortality in both men and women—accounting for about one-third of all deaths in the U.S. Costs related to cardiovascular disease (CVD) place a substantial financial burden on the health care system, accounting for an estimated $320 billion in 2011 [1, 2]. In addition, there is considerable disparity in CVD risk among individuals living in rural settings, particularly medically underserved rural areas and populations [3]. The combination of poverty, environmental factors (such as geographical distances and limited access to healthy foods and physical activity resources), as well as social and cultural attitudes and norms are important contributors to these rural health disparities and collectively compound the problem [4–8].

Another important consideration is gender disparity. Women living in rural areas tend to be uninsured, older, poorer, less educated, and have higher rates of chronic health conditions, and disabilities than their urban counterparts [9]. Rural midlife and older women are often isolated, without access to appropriate physical activity opportunities, affordable healthy food, and healthcare services [6–8, 10–26]. Importantly, women are also 20 % more likely than men to die of heart disease; despite this, many women are unaware that they are at risk for CVD [27]. Fortunately, lifestyle modifications can reduce CVD risk among all age groups, including midlife and older women [28]. Women living in medically underserved areas are a critical target population for CVD prevention efforts. These women can act as powerful role models and agents of change for their families, friends, and communities [29, 30].

There is limited knowledge about how programs and services can move beyond commonly used individual-level approaches, which have limitations in terms of cost, impact, reach, and sustainability, to effectively reduce rural CVD health disparities using an integrated, multi-level, community-engaged approach.

### Program background and development

In 2002, the community-based StrongWomen Strength Training (SWST) program was developed, based upon two decades of clinical and community research that demonstrated the benefits of progressive strength training on midlife and older women’s health—specifically muscular strength and mass, bone health, heart disease, diabetes, frailty, falls, arthritis, depression, and sleep [31, 32]. A national dissemination initiative began in 2003 and there are now approximately 3,000 educators in 48 states trained to implement the SWST classes in their communities, with hundreds of classes operating throughout the U.S. and Canada [33]. In follow-up program evaluations, SWST participants demonstrated improvements in multiple domains of physical fitness (i.e. lower and upper body strength; lower and upper body flexibility; aerobic fitness; and agility) as well as body image and general physical activity behaviors [34, 35].

Ongoing feedback and collaboration with the educators leading those community-based classes catalyzed the development and testing of the StrongWomen Healthy Hearts (SWHH) physical activity and nutrition program. SWHH was tested in a randomized controlled efficacy trial with overweight and obese midlife and older women in Arkansas and Kansas. Results demonstrated significant reductions in body weight and improvements in diet and physical activity behaviors. The program has also been disseminated nationally [36–38].

The most recent addition to the StrongWomen programs is the StrongWomen Change Club (SWCC), which was developed, implemented, and evaluated in 2010–2011. The goal of this program was to promote community-level food and physical activity environment changes in non-urban communities through strategic civic engagement and capacity building activities [39]. One-year follow-up data with eight communities across the U.S. demonstrated the success of residents to identify an issue of concern in their community and work together in a step-wise process to gain broader community support and positively address the issue identified [39].

The Strong Hearts, Healthy Communities (SHHC) program, to be tested in this study, incorporates key elements from these three core StrongWomen curricula as the foundation of a new community-based program targeting CVD risk reduction for rural communities, specifically the strength training and aerobic exercise components from SWST and SWHH, respectively; nutrition education components and behavioral strategies from SWHH; and civic and social engagement strategies and activities from SWCC (also known as and referred to herein as the HEART Club, which is the name now used in this study and others).

Furthermore, to ensure a robust, appropriately tailored intervention, the development of the comprehensive SHHC approach incorporates partnerships with local health educators to conduct community assessments, focus groups, and key informant interviews with members of key groups to gather in-depth information about CVD awareness, economic, healthcare, and social/cultural issues, as well as barriers and facilitators to healthy eating, active living, smoking cessation, stress management, and other relevant topics. In addition, engagement of SHHC participants’ friends and family members in partnered activities and community events is an intervention target, hypothesizing that there will be a reciprocal influence of physical activity and diet behavior between participants and their social network [29, 30].

## Methods/Design

To best support sustainability, a CVD program for rural women living in medically underserved areas should be appropriately tailored and incorporate engagement and capacity building. Thus, the overall objectives of SHHC are to address the gap in knowledge and practice of approaches beyond individual-level change by testing a comprehensive program designed to: a) improve diet and physical activity behaviors, b) promote local built environment resources, and c) shift social norms about active living and healthy eating through civic engagement, capacity building, and community-based programming.

### Specific aims and related approaches

#### Aim 1: Facilitate broad community engagement, build capacity, and conduct formative research; and Aim 1.1: Develop and refine the SHHC curriculum

Community assessments provide an in-depth look at area conditions, characteristics, features, and structures, such as housing, other buildings, schools, public spaces, parks, physical activity facilities, culture/entertainment, street use, commercial activity, signage, media, land use, public transportation, traffic, noise, faith services, health care facilities, community services/organizations, supermarkets, grocery stores, restaurants, and other food venues. The purpose of community assessments for this project is two-fold: 1) To provide community members and researchers with a "360 degree" perspective on the community strengths, resources, needs, and issues of concern; and 2) To develop a "Strong Hearts" resource guide for intervention communities. Information and experiences from the assessment are also important program sustainability tools, given that economic barriers often limit development of new community resources (e.g. parks), and seeing the community through a "new lens" helps identify existing resources that can be improved upon or "marketed." The overall goal of the assessment and resource guides is to address CVD risk factors (e.g. healthy eating, physical activity, preventive services, smoking cessation, stress management). The study team members, including the community health educators, will conduct walking and windshield tours of each community site. The windshield and walking tours are needed to provide detailed contextual information essential to development of the SHHC curriculum, and eventually to facilitate engagement and build capacity within the community.

Focus groups will occur in each of the study communities. We expect responses to key topic areas to vary substantially such that separate groups based on age and gender are justified. Groups will be stratified as such: age groups 40–64 and 65+; and male and female. Focus group participants (8–12 per group) will be recruited and screened by local health educators using a variety of community-based strategies, including press releases, flyers, and website posts. For focus group participants, there are three sources of data: 1) a screening form which will ask about age, BMI, sedentary lifestyle, employment status, and type of health insurance; 2) the focus group discussion, which covers awareness and knowledge about factors related to CVD risk; access to health care services and information; attitudes, perceptions, barriers, and facilitators to physical activity and eating heart-healthy diet; and understanding community in a rural environment; and 3) a short survey to be completed prior to the discussion designed to assess barriers related to attending intervention sessions, medical care seeking behaviors, meal patterns, smoking, and household size and income.

Key informant interviews will occur in study communities, with a total of 30 individuals. The goal is to confirm and extend findings from the community assessments and focus groups, and to provide in-depth perspectives from the communities. Topics will include perceptions of community risk and of environmental, policy, and community social/cultural factors that serve as barriers and facilitators to heart health behaviors. A purposive sample of approximately three key informants per town, representing health educators, practitioners, local leadership, or other stakeholders specific to a community as identified by extension educators will be selected for interviews.

The development of the SHHC and SHHW curricula will be informed by the community assessment, focus group, and key informant interview data and feedback from the local health educator partners along with a systematic content analysis and mapping to fully integrate the three foundational StrongWomen curricula. A national advisory board plus local health educator partners will also provide feedback and input during development of the curricula.

#### Aim 2: Evaluate the efficacy of the SHHC intervention in a 24-week community-based randomized controlled trial; and Aim 2.1: Evaluate changes in behavior, attitudes, and knowledge among participants’ social network

We will evaluate the efficacy of the SHHC intervention on anthropometric, physiologic, behavioral, and psychosocial parameters among overweight and obese women aged 40 and older living in medically underserved rural communities. Sixteen communities, and approximately 12 women per community (N = 192) will be randomized to either SHHC (8 communities) or the Strong Hearts, Healthy Women (SHHW; 8 communities), a minimal intervention control program (described below). There will be six intervention and six control communities in Montana and two intervention and two control communities in New York.

For Aim 2.1, study participants will be asked to identify 1–5 of their closest family members and/or friends, who will then be invited to complete baseline and follow-up questionnaires. To evaluate changes among participants’ social network members, we will collect pre- and post-intervention information about their health behaviors and environment, self-reported height and weight, attitudes and beliefs, and demographic variables.

### Participants

#### Towns/Communities

To be eligible, communities must be classified as Rural–urban Commuting Area (RUCA) code 7 or higher and be designated as a medically underserved area (MUA) or population (MUP) by the Health Resources and Services Administration [40–42]. Within each state, communities are paired and geographically separate to reduce possible contamination effects. Additionally, the median household income of the selected communities must be at least 15 % lower than for the corresponding state [43].

#### Professionals delivering the intervention

The local educators/coordinators who will lead implementation of the intervention, herein referred to as program leaders, will either be county extension educators/agents (http://nifa.usda.gov/extension) or health educators affiliated with the local healthcare system. These program leaders will have extensive experience delivering similar programs to members of their communities. Program leaders and their coordinators will be trained in the study protocol and procedures; they will recruit and screen participants, as well as deliver the program to which they are randomized.

### Recruitment

Program leaders and their coordinators will recruit women via flyers, community bulletin boards, social media, radio, direct mail postcards, and newspapers, as well as through churches, health care providers, human services, and “word of mouth.” Recruitment of Aim 2.1 subjects will occur directly following the Aim 2 subjects’ baseline assessment.

### Screening and eligibility

Potential participants will be screened to ensure that they are in the target population using an Institutional Review Board (IRB)-approved screening form. All women who are eligible based upon initial screening will be required to obtain a signed healthcare authorization form from their healthcare provider indicating that exercise is safe and appropriate before they can begin. Once enrolled, subjects will discontinue the study if there are any changes in medical status that would make exercise unsafe.

#### Inclusion criteria

To qualify, participants must be female, 40 years old or older, have a BMI of 25 or greater, be currently sedentary, and English-speaking. They must also have their physician’s approval and be willing to be randomized to either group. ‘Currently sedentary’ is defined as not meeting Physical Activity Guidelines for Americans or having an estimated total energy expenditure below 34 kcal/kg per day, per the 7-day Physical Activity Recall (PAR).

#### Exclusion criteria

Women will be ineligible to participate if they do not provide informed consent or permission from their healthcare provider, are hypertensive, have a heart rate less than 60 or greater than 100, have cognitive impairment, or are unwilling or unable to complete online questionnaires.

### Intervention curricula

#### Strong hearts, healthy communities

The *Strong Hearts, Healthy Communities* (SHHC) intervention curriculum is the integration of three evidence-informed community programs—two of which target primarily the individual level and a third that targets social and civic engagement. SHHC participants will meet twice per week for hourly sessions for 24 weeks (48 classes), as well as attend out-of-class monthly HEART club meetings (most of which are to be determined and designed by the group). The intervention programmatic components will focus on behavior change in the following areas: physical activity and fitness, weight loss, dietary improvement, and other CVD-related prevention skills and strategies such as stress management [34, 38, 44].

The diet component will include educational elements, aimed at changing dietary patterns informed by DASH (*Dietary Approaches to Stop Hypertension*) diet principles [45–48], the Dietary Guidelines for Americans [49], and the Mediterranean dietary pattern [50] focusing on practical, skill-building activities both in class (e.g. cooking skills, measuring true portion sizes, label reading) as well as field-based learning (e.g. grocery store audits and food environment assessments). The program’s physical activities will be a combination of progressive aerobic exercise and strength training. There will also be out-of-class materials and assignments designed to involve friends and family members in program-related activities [29, 30].

The intervention’s social and civic engagement components will include having SHHC groups work to identify a food or physical activity environment issue they believe is important and feasible to address in their community [39]. This could include adding crosswalks, signage, or bike lanes; it could include creating a healthy after-school or at-work food policy. To support their efforts, and to raise general awareness of local resources for healthy eating and active living, there will also be monthly meetings [39]. SHHC class members will help program leaders plan and implement these events. Example focus areas might include supporting local agriculture and farmers (e.g. healthy local food tasting expo at county fairs); recreation venues and assets for physical activity (e.g. town walk-about at a local park/trail); or health/wellness screening services (e.g. community cholesterol and blood pressure screening).

#### Strong hearts, healthy women

Participants in the minimal intervention program, called Strong Hearts, Healthy Women (SHHW), will meet six times—once per month for an hour for the six month time period. In this program, the nutrition and physical activity content and recommendations will be the same as the SHHC curriculum. The SHHW curriculum does not include a civic engagement component and the participants will not engage in in-class physical activity. It is expected that this minimal curriculum will provide the information to help improve knowledge, but will not provide the same level or amount of social support nor the stimulation for collective impact that SHHC provides.

### Staff training

Program leaders will attend a 1½-day training workshop focused on the general research protocol and will attend a ½ day follow-up intervention training for either SHHC or SHHW directly following randomization. Weekly implementation support calls will be held for all program leaders randomized to SHHC; monthly implementation support calls will be held for all SHHW program leaders.

### Data collection and outcomes

Outcome assessment is planned across anthropometric, physiologic, behavioral, and psychosocial parameters. The study team at Cornell will oversee all online questionnaire-based data collection, including dietary recalls and accelerometry data. An independent agency (Western Health Screening, described below) will travel to the Montana sites to collect the anthropometric and physiologic outcome data; in New York, this will be completed by locally trained staff affiliated with the healthcare system. The schedule for data collection, which occurs at baseline, midpoint (12 weeks), intervention completion (24 weeks) and six-month, one-year, and eighteen months post-intervention, is shown in Table 1. Subjects in Aim 2.1 (family and/or close friends of Aim 2 subjects) will also complete items as indicated in Table 2. All study activities are reviewed and approved by the Cornell IRB (file # 1402004505) and Bassett Medical Center IRB (file #2022).

**Table 1:** Data collection schedule: Aim 2 (SHHC and SHHW participants)

Assessment	Before baseline visit	Baseline visit	Midpoint assessment (12 weeks)	Final assessment (Post-intervention/24 weeks)	Follow-up assessment 1 (6 months post-intervention)	Follow-up assessment 2 (12 months post-intervention)	Follow-up assessment 3 (18 months post intervention)
Informed consent form	X	X					
Demographics	X						
All questionnaires	X			X			X
HBEQ questionnaire only			X		X	X	
Adverse event form			X	X	X	X	X
Midpoint satisfaction survey			X				
Program satisfaction survey				X			
7-day Accelerometer	X		X	X	X		X
7-day 24-hour Dietary recall	X		X	X	X		X
Blood draw		X		X	X		X
Skin scan		X		X	X		X
All anthropometric measurements: Waist and hip circumferences, weight, height (baseline only), body fat, bone density, body composition, blood pressure, and heart rate		X		X	X	X	X
Selected anthropometric measurements: Waist and hip circumferences, weight			X				
Physical function tests (arm curl, chair, two minute step)		X	X	X	X		X

**Table 2:** Data collection schedule: Aim 2.1 (Social network members)

Assessment	Baseline assessment	Final assessment (Post-intervention/24 weeks)	Follow-up assessment (6-months post-intervention)
Informed consent form	X		
Demographics	X		
Self-reported height and weight (Height at baseline only)	X	X	X
Self-reported physical activity	X	X	X
Self-reported diet	X	X	X
Attitudes and beliefs toward healthy eating and physical activity	X	X	X

#### Measures

##### Simple 7 and Framingham risk score

Simple 7 is a cardiovascular health metric comprised of four health behaviors (i.e. smoking, body mass index, physical activity, healthy diet) and three health factors (i.e. total cholesterol, blood pressure, fasting glucose) [51, 52]. They are characterized on a scale of poor, intermediate, or ideal health, which is correlated with prevalence of CVD events [53, 54]. We will use this approach to determine a Simple 7 cardiovascular health score at baseline and all post-intervention time points [54]. The Framingham Risk Score will also be calculated from questionnaire and physiologic data at baseline and post-intervention [55, 56]. Age, smoking status, total cholesterol, HDL cholesterol, and systolic blood pressure are used to calculate the Framingham Risk Score.

##### Health behaviors environment, and quality of life

This comprehensive questionnaire includes items related to nutrition and physical activity behaviors; environmental and social factors; other health behaviors (e.g. smoking); and limitations of activities due to health [4, 5, 57–65]. In addition, physical activity and sedentary behaviors will be measured using the 7-item International Physical Activity Questionnaire [66] and fruit and vegetable intake will be measured using the National Cancer Institute’s Fruit and Vegetable Questionnaire [67].

##### Social support and self-efficacy

Social support and self-efficacy for physical activity and diet will be measured using adapted versions of the Sallis tools [68–72].

##### Depression anxiety, stress, and resilience

Depressive symptoms will be measured using the 8-item Patient Health Questionnaire (PHQ-8) [73]. The 7-item Generalized Anxiety Disorder scale (GAD-7) will be used to measure anxiety [74]. Stress will be measured using the 10-item Perceived Stress Scale (PSS) [75–77]. Resilience will be measured using the Brief Resilience Scale [78].

##### Eating behaviors

The 21-item Three Factor Eating Questionnaire (TFEQ-R21) will be used to measure three eating behaviors: cognitive restraint, uncontrolled eating, and emotional eating [79–83].

##### Demographic variables (baseline only)

Program leaders and all SHHC subjects will complete a questionnaire that includes basic demographic variables (e.g. age, race/ethnicity, education, income, household size). Questions will be derived from national surveys (e.g. U.S. Census).

#### Anthropometric and physiologic measures

Anthropometric and physiologic data, including blood draws, will be collected by Western Health Screening (WHS) in Montana and by locally trained staff affiliated with the healthcare system in New York, with logistical support from program leaders and the study team. Blood will be drawn by trained, experienced phlebotomists. Anthropometric, physiologic, and dermal measures will all be taken at baseline and outcome assessments. For anthropometric and physiologic data, weight, hip circumference, and waist circumference measurements will be taken at the midpoint assessment (12 weeks).

##### Anthropometric measures

These measures will include height, weight, BMI, body fat, bone density, hip circumference, waist circumference, and body composition. Freestanding height boards will be used for height measurements, and balanced scales will be used for weight calculations. The Omron HBF-306 will be used to measure body composition by electrical impendence. The Achilles Express and Insight will be used to measure bone density. A retractable Gulick tape measure will be used for hip and waist circumferences, rounded to the nearest 0.125 inch. Height, weight, and hip and waist circumferences will be measured in duplicate, unless specified criteria are not met for the two measurements. In that case, a third measurement will be taken. These anthropometric measurements are primary outcomes for the study.

##### Physiologic measures

These measures will include blood pressure, resting heart rate, and fasting blood draws to assess 12-hour fasting glucose, hemoglobin A1C, C-reactive protein, and lipid panel including direct LDL cholesterol, total cholesterol, HDL cholesterol, cholesterol/HDL ratio, and triglycerides [84].

##### Dermal measures

As an objective measure of fruit and vegetable intake, study staff will use a Pharmanex BioPhotonic Scanner at baseline and outcome assessments, which non-invasively measures carotenoid levels in skin tissues using Raman Spectroscopy [85].

#### Physical activity, diet, and functional fitness measures

Measures of physical activity, diet, and functional fitness are secondary outcomes of the study.

##### Physical activity

Objective measurement of physical activity will be obtained using the ActiGraph Model GT3XE accelerometers worn for seven days at baseline, midpoint (12 weeks), outcome (24 weeks), one year, and eighteen months.

##### Diet

Dietary and supplement/vitamin intake will be collected and analyzed using seven automated self-administered 24-hour dietary recalls (ASA-24) [86]. At least one weekend day recall will be collected and at least three weekday recalls will be collected at all study time points.

##### Functional fitness

The chair test, bicep (arm curl) test, and 2-minute step test will follow the Senior Fitness Test protocol and be completed at baseline, 12-week, outcome (24 weeks), six months, and eighteen months [87]. The chair test and arm curl test consist of counting stands and arm curls completed in 30 seconds [87]. The 2-minute step test evaluates the number of times stepped in two minutes [87].

#### Process evaluation

##### Leader and participant-level process evaluation

Leaders will complete questionnaires after each class session related to attendance, as well as program delivery and fidelity [88, 89], and for SHHC sites they will report on subjects’ participation in HEART Club meetings and related activities. All SHHC participants will be provided with a Fitbit, a wireless activity tracker worn on the wrist, to enhance self-monitoring; they will be asked to share their Fitbit data with the study and that data along with the participant logs will provide on-going reported and objectively measured physical activity data. Participants will also be asked to complete a civic engagement questionnaire designed to assess awareness of local resources and civic engagement participation (past and current). Civic engagement attitudes and behaviors will be measured using the Civic Engagement Scale [90]. The questionnaire will be administered to all subjects at baseline and post-intervention.

##### Economic evaluation

Standard economic evaluation methods will be used to compare the value of the resources used in the SHHC project to the health consequences. Information on salaries, wages and benefits; cost of facilities (office space and utilities); equipment, supplies, and travel; and staff training will be collected from program leaders. Information on time costs (participants’ time at hourly wage rate), travel costs, and time spent exercising and planning/preparing meals will be collected from participants.

### Randomization

The study statistician will determine randomization assignment based upon a matched RUCA and region classification such that, for paired towns, one will be randomized to SHHC and the other will be randomized to SHHW. Following baseline assessments, town randomization assignments will be revealed to program leaders and subjects.

### Data management and analytic plan

#### Sample size calculations

Based upon the most recent findings from the SWHH study [38], in which participants lost 2.1 kilograms (SD = 2.6) over twelve weeks, it was determined that a sample size of 34 people per group will allow us to detect an effect size of 0.690 with a 2-sided test and a power of 80 %, conservatively allowing for a standard deviation of 3. Given that the data are clustered within counties, we also assumed intra-class correlation of 0.025 (with clusters of 12 people) and 10 % attrition [88], yielding a sample size requirement of 48 people per group (96 total) to obtain 80 % power. This sample size will also allow us to have sufficient power to detect an effect size of 0.690 among secondary outcomes, such as blood pressure. For example, based on prior exercise intervention research with overweight and obese midlife and older women [91], an effect size of 0.690 would correspond to a 10 % difference in systolic blood pressure with a standard deviation of approximately 24.

#### Quantitative analysis

Data will be collected online or double-entered into SPSS Data Builder by trained research personnel when needed. Univariate descriptive statistics for all variables will be examined. Problematic cases with outliers will be investigated and possibly rectified. Descriptive statistics by treatment groups will be compiled and tabulated. Comparison between conditions will be completed using chi-square test (binary and categorical variables), *t*-test (continuous variables), or non-parametric Wilcoxon Signed-rank test (continuous variables unsuitable for *t*-test). Since the observations are clustered within communities, we will use multi-level linear regression models to examine the unadjusted and adjusted effects of the intervention on the primary outcome (change in body weight) and secondary outcomes [physiologic (e.g. blood pressure, lipids, c-reactive protein, hemoglobin A1C); anthropometric (e.g. waist circumference); behavioral (e.g. 7-day accelerometry); and psychosocial (e.g. quality of life)] parameters using *intent-to-treat analysis*. The community will be entered in the model as a random effect. Adjusted models will control for baseline values of the outcome, age, education level, marital status, smoking status, and other relevant covariates as fixed effects in addition to the treatment variable. Interactions between the treatment variable and other covariates will also be tested. Potential mediating effects of behavior, psychosocial, and community awareness/participation variables will be examined. In addition to the direct effect of the intervention on the primary outcome, the indirect effects of the intervention on anthropometric and physiologic outcomes through behavior, psychosocial, and community awareness/participation outcomes will be investigated. Multiple regression and/or structural equation modeling will be used to assess the contribution of various indirect and the direct effect of the intervention.

To address Aim 2, change variables for outcomes of interest (e.g. BMI) will be created by subtracting the measurement at 24 weeks from the corresponding baseline measure. A multilevel model will be used, as the observations are nested within communities. To account for the non-independence between observations from members of the same town, workplace, or household, a community and workplace/household ID will be entered in the model as a random effect. Intervention will be entered as a fixed independent variable. The model will control for baseline values of the outcome, age, education, marital status, smoking status, and other relevant covariates as fixed effects. Interaction terms will also be tested.

#### Economic analysis

Standard economic evaluation methods will be used to compare the value of the resources used in the SHHC project to the health consequences. As a first step, a cost analysis to identify and measure the direct, tangible costs of the resources used in program administration and implementation will be conducted. Important cost categories are salaries, wages, and benefits; facilities (office space and utilities); equipment, supplies, travel, and staff training. The cost analysis will be conducted from the narrow program perspective and from the broad societal perspective. For the program perspective, the focus will be on costs directly incurred by the agencies that administer and implement the program. These costs will include both direct payments and the value of in-kind contributions, such as the value of contributed office space. The results of the program-perspective cost analysis will provide information to judge whether and where the SHHC can be disseminated. For this purpose, it will be important to distinguish the costs of different components of the SHHC, for example, the costs of SHHC program development as distinct from the costs of the SHHC intervention. The detailed results will allow groups considering dissemination to develop cost predictions tailored to their specific context.

A preliminary cost-effectiveness analysis (CEA) of the SHHC intervention will be conducted. The CEA will build on the cost analysis conducted from the broad societal perspective. From the societal perspective, costs include not only the costs included in the program-perspective cost analysis, but also the opportunity cost of all resources used as a result of the intervention. Costs to participants are an important cost of the opportunity costs included from the societal perspective. Participants give up time that could have been used in other valued ways such as labor market work, household work, or leisure activities. Standard practice of measuring the value of participants’ time based on the relevant wage rates will be followed. The incremental costs of the SHHC intervention will be compared to the incremental effectiveness estimated from the controlled trial. The incremental cost-effectiveness ratio (ICER) will be calculated by dividing the incremental costs of the intervention by the incremental effectiveness. The calculated ICER in terms of the primary outcome will provide an estimate of the costs per unit change in body weight. This ICER allows the direct comparison of the cost-effectiveness of the SHHC intervention to alternative approaches to reduce body weight. The ICER will also be calculated in terms of standardized health outcomes including life years and quality-adjusted life years (QALYs). Epidemiologic models will be used to map the effects measured in the controlled trial (body weight, physiologic measures) to predict impact on life years and QALYs. The calculated ICER in terms of QALYS will allow the cost-effectiveness of the SHHC to be compared to a wide range of other health interventions for which QALY-based CEAs have been performed. An important goal of the preliminary CEA is to demonstrate feasibility and identify important issues to be addressed in more complete economic analyses.

## Discussion

There are notable disparities in risk for obesity, hypertension, diabetes, and CVD for people living in rural settings, particularly underserved rural areas. These disparities are driven by complex factors such as socioeconomic disadvantage, geographical distances/barriers, social and cultural issues, and limited access to healthcare, healthy foods, and/or physical activity opportunities due to environmental constraints, affordability, and availability. Moving beyond individual-level programs toward integrated, multi-level, community-engaged approaches may more effectively reduce rural CVD health disparities.

The novel integration of a multi-level, community-informed program combined with civic engagement and capacity building focused on local resource awareness and enhancement has the potential to effect clinically meaningful improvements among participants, as well as their families, friends, and communities. This innovative approach will also help sustain positive changes by linking behavior, social support, and the community environment. If successful, SHHC could be nationally disseminated, providing a feasible model for underserved rural communities across the nation to improve health, well-being, and quality of life and reduce CVD and other chronic diseases.

## Abbreviations

ACSM: American College of Sports Medicine
ALB: Albumin
ALT: Alanine aminotransferase
AST: Aspartate aminotransferase
ASA-24: Automated Self-administered 24-hour Recall
BMI: Body mass index
BUN: Blood urea nitrogen
CEA: Cost-effectiveness analysis
CVD: Cardiovascular disease
DASH: Dietary approaches to stop hypertension
EDTA: Ethylenediaminetetraacetic acid
GAD-7: Generalized anxiety disorder screener
GGT: Gamma-glutamyltransferase
HBEQ: Health behavior and environment questionnaire
HDL: High-density lipoprotein
IBC: Iron binding capacity
ICER: Incremental cost-effectiveness ratio
IRB: Internal review board
LDH: Lactate dehydrogenase
LDL: Low-density lipoprotein
MUA: Medically underserved area
MUP: Medically underserved population
PAR: Physical activity recall
PHQ-8: Patient health questionnaire depression scale
QALY: Quality adjusted life years
RUCA: Rural urban continuum code
SHHC: Strong hearts, healthy communities
SWCC: StrongWomen change club
SHHW: Strong hearts, healthy women
SWHH: StrongWomen healthy hearts
SPSS: Statistical package for the social sciences
SWST: StrongWomen strength training
TFEQ-R21: Three factor eating questionnaire
TSH: Thyroid stimulating hormone
WHS: Western health screening
